# Synchronous Diagnosis of Ductal Carcinoma In Situ and Mucoepidermoid Carcinoma of the Parotid Gland: A Rare Case of Dual Primary Malignancy

**DOI:** 10.7759/cureus.87180

**Published:** 2025-07-02

**Authors:** Hesa A Salaibeekh, Hesa Alkaabi

**Affiliations:** 1 Family Medicine, Bahrain Defense Force, Riffa, BHR

**Keywords:** ductal carcinoma in situ (dcis), mucoepidermoid carcinoma (mec), multiple primary malignancies, parotid gland, salivary gland tumor

## Abstract

The coexistence of two histologically and anatomically distinct malignancies, ductal carcinoma in situ (DCIS) and mucoepidermoid carcinoma (MEC), in the same patient is a clinically rare phenomenon. MECs of the salivary glands, most often arising in the parotid, can display a range of histological grades and clinical courses. We report the case of a 76-year-old woman with a prior diagnosis of hormone receptor-positive DCIS who developed low-grade MEC of the parotid gland nearly a decade later. Possible etiologic factors discussed include coincidental tumor development, prior treatment effects, inherited predisposition, and hormonal influences. A focused literature-based discussion supports the analysis of this unique case.

## Introduction

The synchronous occurrence of two distinct primary malignancies, ductal carcinoma in situ (DCIS) of the breast and mucoepidermoid carcinoma (MEC) of the salivary gland, is a clinically rare phenomenon. This report describes a unique case involving a 76-year-old woman with a prior diagnosis of hormone receptor-positive DCIS who developed low-grade MEC of the parotid gland nearly a decade later.

DCIS is a non-invasive neoplastic proliferation of epithelial cells within the breast ducts, often asymptomatic and detected via routine screening mammography [1]. With the rising use of mammographic screening, the incidence of DCIS has increased significantly, and it now comprises up to 25% of newly diagnosed breast neoplasms in the Western world [2,3]. Management commonly includes breast-conserving surgery, radiotherapy, and adjuvant endocrine therapy for hormone receptor-positive tumors [4]. Long-term survival for DCIS exceeds 95%, and while recurrence can occur, distant metastases are rare in the absence of invasion [5].

MEC is the most frequently encountered malignant salivary gland neoplasm, accounting for roughly one-third of all cases, and arises primarily within the parotid gland [3,6]. It consists of mucin-producing, intermediate, and squamous epithelial cells and demonstrates diverse histopathological grades, which predict prognosis [7]. Low-grade MECs typically have favorable outcomes following surgical resection, while high-grade tumors may behave aggressively [6,8].

The occurrence of multiple primary malignancies, although uncommon, has been documented in cancer registries. Studies report that up to 10% of cancer survivors may develop a second primary cancer, particularly in the elderly population, where cumulative mutational burden, immune senescence, and longer post-treatment survival contribute to risk [9,10]. However, the co-presentation of breast DCIS and salivary MEC (tumors with disparate etiologies and anatomic origins) has not been reported in large series and thus warrants further exploration.

## Case presentation

A 76-year-old Bahraini woman was diagnosed in 2014 with right breast DCIS after presenting with a palpable mass. Histopathological analysis confirmed grade 2 DCIS with HER2 overexpression (3+) and estrogen receptor (ER) and progesterone receptor (PR) positivity. Initial staging classified the disease as T1N1M0. She underwent breast-conserving surgery with negative margins and sentinel lymph node biopsy, followed by four cycles of adjuvant chemotherapy with epirubicin and cyclophosphamide (EC), and five years of letrozole. She did not receive radiotherapy due to favorable surgical margins and absence of invasive disease. Her surveillance mammograms remained unremarkable through April 2022.

In September 2023, she presented with a progressively enlarging, painless swelling in the left periorbital and preauricular region. Initial clinical suspicion was sialadenitis, and she received multiple empirical antibiotic courses without clinical improvement. Examination revealed a firm, mobile mass overlying the left parotid gland, without facial nerve involvement. There were no systemic signs such as fever, weight loss, or night sweats.

Neck ultrasonography demonstrated a heterogeneous, hypoechoic lesion within the superficial lobe of the left parotid gland. Fine-needle aspiration cytology revealed clusters of mucin-secreting and intermediate cells, with immunohistochemical positivity for CK7 and CK5/6, and strong mucicarmine staining. MRI confirmed a well-demarcated 2.2 cm lesion localized to the superficial parotid with no lymphadenopathy or perineural spread.

**Figure 1:**
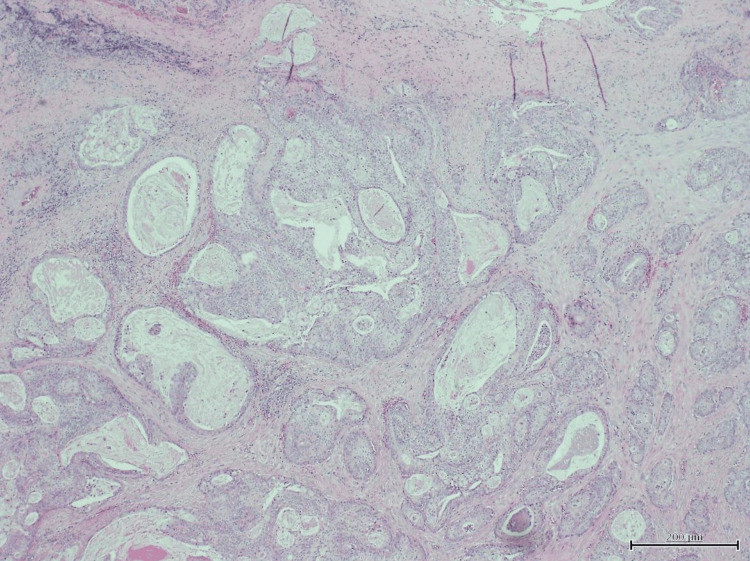
Hematoxylin and eosin (H&E) stain, low-power view at 10x magnification showing infiltration by nests of tumor cells, some containing intraluminal secretions, within a fibroinflammatory stroma

**Figure 2:**
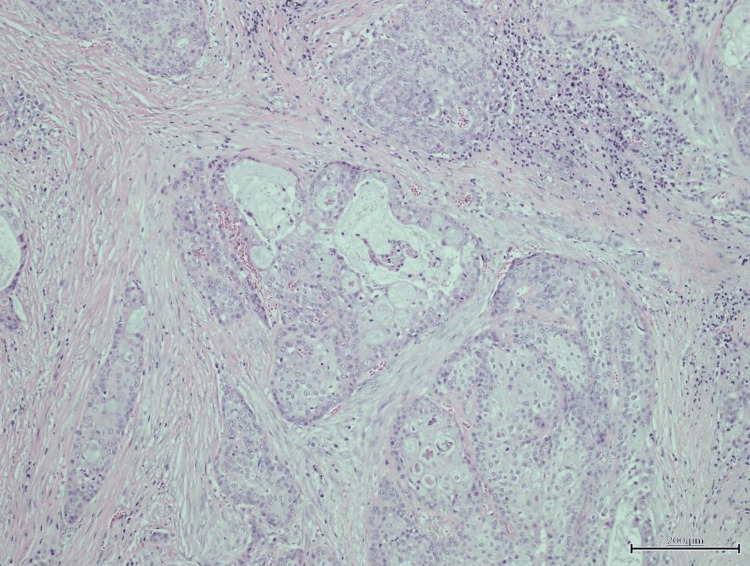
Hematoxylin and eosin (H&E) stain, high-power view at 40x magnification showing infiltrative tumor cells composed of mucous cells, intermediate cells, and squamous cells, surrounded by desmoplastic stroma

A superficial parotidectomy was performed with preservation of the facial nerve. Histopathological examination confirmed low-grade mucoepidermoid carcinoma, with negative margins and no lymphovascular invasion (Figures 1, 2). The patient’s recovery was uneventful, and she remains on oncologic follow-up.

## Discussion

The concurrence of DCIS and MEC, two malignancies without overlapping histological lineage or anatomical continuity, prompts a multifaceted discussion. DCIS arises from ductal epithelial cells of the breast and is largely confined to the ductal system, while MEC originates in the salivary gland ductal reserve cells and demonstrates mucous and epidermoid differentiation [3,6].

The most plausible explanation is a coincidental occurrence of two primary malignancies. The incidence of multiple primaries in cancer survivors ranges between 4-10%, especially in patients over 70 years, as shown in Surveillance, Epidemiology, and End Results (SEER) data [9,11]. Dual cancers with no embryologic, hormonal, or treatment-related links are often reported in the literature as sporadic findings [10].

A secondary consideration is radiation exposure. Ionizing radiation has been conclusively linked to salivary gland carcinogenesis, particularly after therapeutic or environmental exposure during childhood [12]. Nevertheless, our patient did not receive radiotherapy to the breast or any region close to the head and neck. In modern radiotherapy protocols for breast cancer, scatter doses to distant tissues such as the parotid are negligible [13]. Thus, radiation-induced MEC is implausible in this case.

Genetic predisposition to multiple cancers must also be considered. Syndromes such as Li-Fraumeni (TP53 mutations), Cowden syndrome (PTEN mutations), and hereditary breast and ovarian cancer syndrome (BRCA1/2 mutations) have all been implicated in predisposition to multiple tumors [14,15]. However, MEC is not commonly associated with these syndromes, and our patient’s personal and family history was not indicative of a hereditary cancer syndrome. Additionally, MECs frequently harbor MAML2 fusions, which are not implicated in breast carcinogenesis [16].

Hormonal influences could also be hypothesized, given the hormone receptor positivity in DCIS and the patient’s exposure to systemic endocrine therapy. However, while androgen receptor expression has been noted in some salivary gland malignancies such as salivary duct carcinoma, there is scant evidence of ER or PR playing a role in MEC pathogenesis [17]. Williams et al. found that hormone receptors are variably expressed across salivary gland carcinomas, but MECs rarely express ER or PR in a functionally relevant manner [17]. Therefore, any hormonal link is weak and unsubstantiated.

Finally, the case highlights the clinical importance of maintaining diagnostic breadth in cancer survivors. Misattributing new symptoms to benign processes, such as infection or recurrence, can delay diagnosis. As in this patient, early biopsy and imaging of new lesions are essential to distinguish between recurrence, metastasis, or new primary tumors.

## Conclusions

This report presents an unusual case of sequential DCIS and MEC in the absence of radiation exposure or hereditary cancer syndrome. While coincidence remains the most likely explanation, the case underscores the importance of comprehensive diagnostic evaluation in cancer survivors presenting with new symptoms. Ongoing advances in molecular oncology may eventually elucidate subtle links between disparate tumor types, but current evidence supports independent tumorigenesis in this case.
